# Low molecular weight heparin-induced thrombocytopenia management during hemodialysis and cardiac surgery: a case report and literature review

**DOI:** 10.1186/s40981-025-00781-0

**Published:** 2025-03-28

**Authors:** Shuto Takada, Shogo Suzuki, Takahiro Tamura

**Affiliations:** 1 Department of Anesthesiology, Nagoya University Hospital, Nagoya, Japan; 2 Department of Anesthesiology, Nagoya University Graduate School of Medicine, 65 Tsurumai-Cho, Showa-Ku, Nagoya, 466-8550 Japan

**Keywords:** Dalteparin, Heparin-induced thrombocytopenia, Low molecular weight heparin

## Abstract

**Background:**

Heparin-induced thrombocytopenia (HIT) is a serious complication of heparin therapy, including low molecular weight heparins (LMWHs) like dalteparin. While LMWHs reduces the risk of HIT compared to unfractionated heparin, vigilance remains essential.

**Case presentation:**

An 82-year-old male with chronic kidney disease (CKD) developed HIT during hemodialysis anticoagulation with dalteparin, resulting in a platelet count of 17,000/µL and positive HIT antibodies. Dalteparin was replaced with nafamostat mesilate. Following confirmed HIT antibody seronegativity, elective aortic valve replacement was performed under cardiopulmonary bypass using heparin. Postoperative dialysis was managed using nafamostat mesilate, preventing HIT recurrence. His platelet count recovered after dalteparin replacement, and no recurrence of HIT was observed.

**Conclusions:**

Even LMWHs, such as dalteparin, pose a HIT risk, necessitating vigilant monitoring. Confirming HIT antibody seronegativity and appropriately timing surgery are critical for patients with a history of HIT. Proper postoperative follow-up and alternative anticoagulation strategies can prevent HIT recurrence.

## Background

Heparin is an anticoagulant that is widely used to treat and prevent thromboembolism, prevent blood clotting while using extracorporeal circulation devices, including during hemodialysis and cardiopulmonary bypass, and prevent blood clotting during vascular catheter insertion. Heparin-induced thrombocytopenia (HIT) develops in some cases; exposure to heparin causes thrombocytopenia and thrombus formation through platelet factor 4-heparin complex antibodies (HIT antibodies) [1]. Low molecular weight heparin (LMWH) is said to reduce the risk of HIT development by approximately 10% compared to unfractionated heparin [2]. Here, we report a case in which HIT developed during hemodialysis anticoagulation management with dalteparin, a LMWH. After HIT antibody seronegativity, elective aortic valve replacement was performed under cardiopulmonary bypass using heparin. Subsequently, postoperative dialysis was managed with nafamostat mesilate, with no recurrence of HIT.

## Case presentation

Consent was obtained from an 82-year-old male (160 cm, 48 kg) with hypertension, hyperlipidemia, CKD, and a nonfunctioning left adrenal mass. Medications included carvedilol 2.5 mg, azosemide 30 mg, pitavastatin calcium 4 mg, lanthanum carbonate 2250 mg, lactulose 18 mg, and darbepoetin 30 µg/week. In year X-2, he was diagnosed with moderate aortic valve stenosis (AS) with a left ventricular ejection fraction (EF) of 60%, aortic valve area (AVA) of 1.30 cm^2^, and a maximum blood flow velocity of 3.14 m/s. In January of year X, hemodialysis was initiated, conducted three times a week for four hours each, using dalteparin according to the institution's protocols due to worsening CKD. Post-hemodialysis, he experienced shortness of breath, elevated brain natriuretic peptide, and worsening heart failure due to volume overload. Echocardiography showed worsening AS with 30% EF, 3.22 m/s maximum blood flow velocity, and 1.10 cm^2^ AVA. Aortic valve replacement was planned; however, it was postponed due to a drop in platelet count to 17,000/µL and the presence of positive HIT antibodies, although anti-platelet antibodies were negative. During this time, no thrombosis was observed in the dialysis circuit. Dalteparin was replaced with nafamostat mesilate. Additionally, due to concerns about drug-induced thrombocytopenia, the new medication was subsequently discontinued. By May of year X (46 days after the positive HIT antibody test), the patient was referred to our hospital, where the patient tested negative for HIT antibody, and the platelet count increased to 159,000/µL. In August of year X (122 days after the positive HIT antibody test), aortic valve replacement and mitral valve repair were planned with heparin during intraoperative cardiopulmonary bypass, scheduled at the convenience of both the patient and the surgeon.

### Preoperative findings

A chest radiograph showed a cardiothoracic ratio of 66%. An electrocardiogram revealed sinus rhythm and left bundle branch block. Transesophageal echocardiography showed a diffuse reduction in left ventricular wall motion with 20% EF. The aortic valve was tricuspid, with calcification on the right coronary and non-coronary cusps and severe aortic stenosis with an AVA of 0.77 cm^2^. Moderate mitral and tricuspid regurgitation were also observed. Coronary angiography showed stenosis in the left coronary artery #9 = 90%, #12 = 75%, and #14 = 75%. Blood tests showed 11.6 g/dL hemoglobin, 116 × 10^4^/μL platelet count, 1.09 prothrombin time-international normalized ratio, 1.1 activated partial thromboplastin time ratio, 381 mg/dL fibrinogen, and 82.1% antithrombin III.

### Clinical and anesthesia course

After the necessary induction of anesthesia, the operation commenced, and the chest was opened via sternotomy, and 16,000 units of sodium heparin were administered. The activated clotting time was confirmed to be extended to 478 s, and extracorporeal circulation was established. Aortic valve replacement and mitral valve repair were performed. After weaning from cardiopulmonary bypass, 150 mg of protamine sulfate antagonized heparin. The chest was then closed without any major complications, and the operation was completed. The operation lasted 4 h and 36 min, with 2 h and 9 min of cardiopulmonary bypass. Blood loss was 1860 mL, and transfusion included 4 units of fresh frozen plasma, 12 units of cryoprecipitate, and 20 units of platelets.

### Postoperative course

**Fig. 1:**
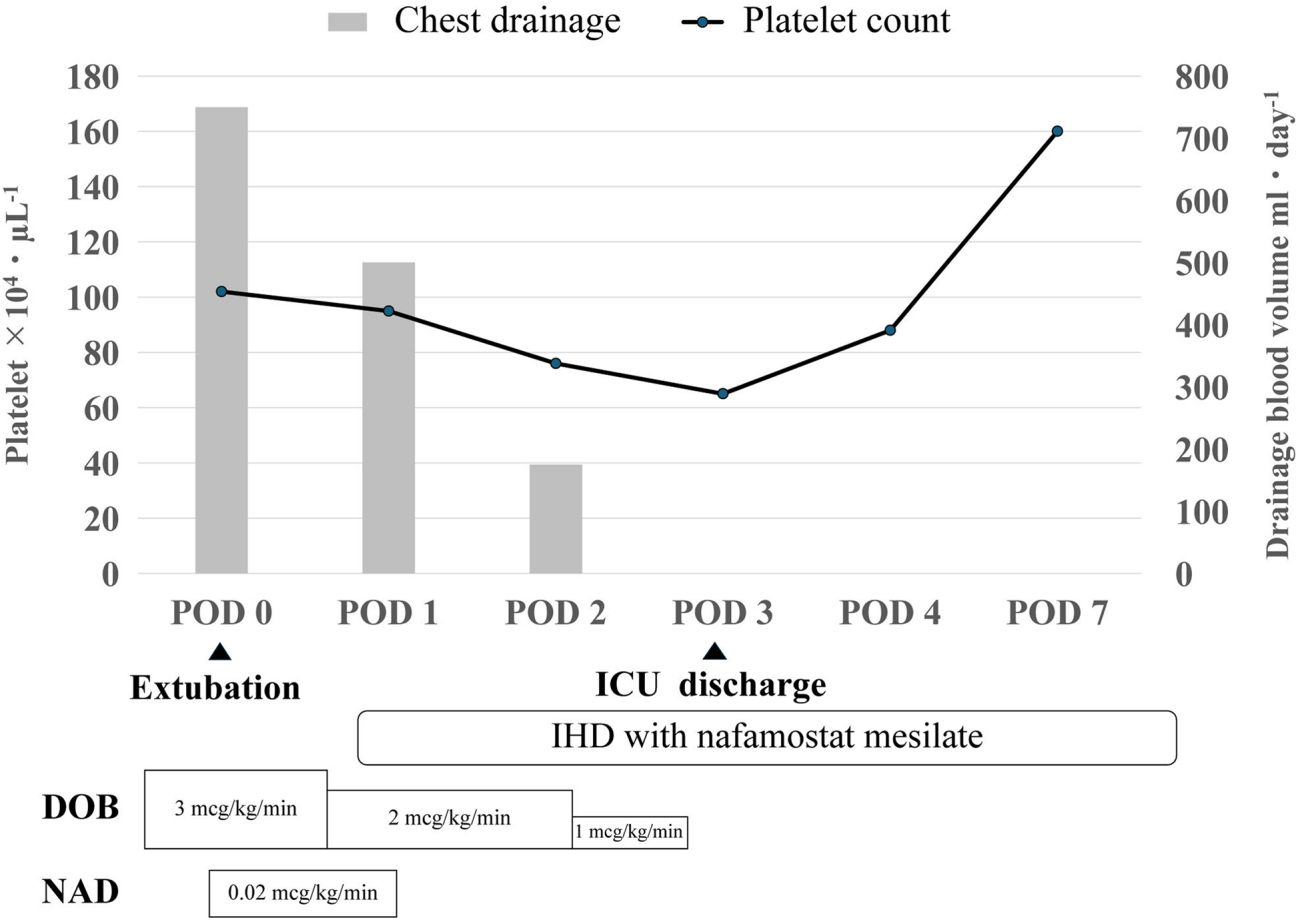
Postoperative course. Although there was a temporary drop in platelet count after surgery, the bleeding subsided, and the patient was observed without the need for transfusion. The platelet count began to improve from POD 4. POD; Post-operative day, ICU; Intensive care unit, IHD; Intermittent hemodialysis, DOB; Dobutamine, NAD; Norepinephrine

The patient was stable in the intensive care unit and was extubated on the day of surgery. Intermittent dialysis resumed with nafamostat mesilate starting on the first postoperative day (Fig. 1). The platelet count continued decreasing until the third postoperative day without any bleeding. The patient was discharged from the intensive care unit on the third day. From day 4, the platelet count started increasing, and he was discharged from the hospital on the 28th day post-surgery with no recurrence of HIT.

## Discussion

In this case, HIT was caused by dalteparin used as an anticoagulant for hemodialysis. LMWHs, derived from unfractionated heparin, differ chemically and pharmacokinetically [3, 4]. Their molecular, structural, physicochemical, and biological properties vary [5]; hence, doses are not interchangeable and should be based on proven clinical safety and efficacy [6]. While LMWHs reduce the risk of HIT by approximately 10% compared to unfractionated heparin [2], the risk varies among different LMWHs. This reduced risk is partly attributed to the fact that, due to its smaller molecular size, LMWH does not interact with PF4 and platelets as effectively as UFH. Furthermore, HIT antibodies induced by LMWH are more frequently of the IgA and IgM class, while UFH-induced HIT antibodies are more often of the IgG class, which is more strongly associated with clinical HIT [7, 8].

Regarding reports of HIT caused by dalteparin, we reviewed 15 articles obtained from PubMed using the Medical Subject Headings (MeSH) terms "dalteparin/adverse effects" and "thrombocytopenia/chemically induced" on January 1, 2024, and found four articles that met the criteria [9–12] (Table 1); in these articles, HIT developed 30 days after starting dalteparin in one case and 8–14 days in others. In our case, HIT developed gradually over more than 60 days.

**Table 1:** Cases of HIT induced by dalteparin sodium reported in the literature

Author	Age/Sex	Medical history	Purpose and dose	Onset (Days)	Differential diagnosis
Giorgi et al	82/F	Multiple myeloma AKI	Anticoagulant for dialysis 5000 U/day	14	Chemotherapy
Tvito et al	85/F	Hip fracture	Thromboprophylaxis 5000 U/day	8	ITP
Lovatt et al	41/F	Pregnancy DVT	Thromboprophylaxis 18,000 U/day	12	-
Gan et al	83/F	Hip fracture	Thromboprophylaxis 5000 U/day	30	-

*HIT* Heparin-induced thrombocytopenia, *AKI* Acute kidney injury, *DVT* Deep vein thrombosis, *ITP* Immune thrombocytopenia

A literature review on cardiac surgery following HIT onset due to dalteparin revealed no relevant reports among the 513 articles obtained from PubMed using the MeSH terms "cardiac surgery" and "thrombocytopenia/chemically induced (search: February 15, 2023)."

Dialysis HIT typically occurs during heparin dialysis initiation. Early symptoms include unexplained blood clots in the circuit [13]; however, no thrombosis was observed in the present case. Blood clots in the circuit with no other suspected cause aside from HIT are pathologically equivalent to thrombus but do not fall under the category of arterial or venous thrombosis (a disease name that adds 2 points to the T's score for thrombosis), so the score for blood clots in the circuit is 1 point [14]. The 4 T's score for intermittent dialysis may be lower than the actual score due to different thrombocytopenia rates and timing. Thrombocytopenia in intermittent dialysis often emerges during the 5th or 6th session, necessitating broad timing considerations from 7 to 30 days [15].

This patient’s 4 T score was 4 points (1 point for thrombocytopenia, 1 point for the onset time, and 2 points for other causes of thrombocytopenia), according to the attending clinician’s judgment. Given the positive HIT antibodies and the absence of other diseases suggesting obvious thrombocytopenia, the patient was diagnosed with clinical HIT, resulting in postponed surgery. Functional assays, although not performed here, are not widely used in Japan. Warkentin et al. [14] proposed the tip of the iceberg model [16], wherein 30% of patients with positive HIT antibodies due to unfractionated heparin, functional tests were positive in about 10%, HIT occurred in 5%, and HIT accompanied by thrombosis occurred in about 4%. Therefore, diagnosing HIT based solely on positive antibodies can lead to overdiagnosis; however, in this case, the HIT diagnosis was corroborated by a platelet count recovery post-nafamostat.

A major ICU management concern was HIT recurrence. Antibodies typically become undetectable around 100 days post-HIT onset, and surgery was performed over 4 months later. Publications report successful heparin re-administration post-HIT antibody conversion without recurrence [17–20]. The recurrence risk from heparin re-exposure during surgery in patients with a history of HIT is low, similar to that of patients without a history of HIT. Preventive measures, such as avoiding the use of a full course of heparin, should be taken [21]. We also avoided heparin-coated central venous catheters and heparin for arterial pressure management (our hospital is heparin-free in daily practice [22], and we performed dialysis management using nafamostat mesilate, which was also used in preoperative dialysis anticoagulation management, limiting the use of heparin to cardiopulmonary bypass only). Since there was a possibility that HIT would develop again due to the re-administration of heparin, clinical symptoms and test results, such as platelet count, were carefully observed after surgery. Fortunately, there was no thrombocytopenia or embolism, and it was determined that there would be no recurrence of HIT, and the patient's postoperative course was uneventful.

In conclusion, while LMWHs reduce HIT risk compared to unfractionated heparin, the possibility of HIT must always be considered, and careful follow-up is required when using heparin preparations, including LMWH. For cardiac surgery in patients with HIT history, confirming negative HIT antibodies, selecting appropriate timing, minimizing heparin use, and conducting post-surgery monitoring should be performed. Interpreting the usual 4 T's score in dialysis patients demands comprehensive judgment based on clinical and immunological findings.

## Data Availability

Not applicable.

## Abbreviations

AS: Aortic valve stenosis
AVA: Aortic valve area
CKD: Chronic kidney disease
EF: Ejection fraction
HIT: Heparin-induced thrombocytopenia
LMWH: Low molecular weight heparin
